# Fluid Flow Stimulation Modulates Expression of S100 Genes in Normal Breast Epithelium and Breast Cancer

**DOI:** 10.1007/s12195-021-00704-w

**Published:** 2021-10-27

**Authors:** Kenneth F. Fuh, Jessica Withell, Robert D. Shepherd, Kristina D. Rinker

**Affiliations:** 1 Biomedical Engineering Graduate Program, University of Calgary, Calgary, AB T2N 1N4 Canada; 2 Cellular and Molecular Bioengineering Research Lab, University of Calgary, Calgary, AB T2N 1N4 Canada; 3 Department of Chemical and Petroleum Engineering, University of Calgary, Calgary, AB T2N 1N4 Canada; 4 Arnie Charbonneau Cancer Institute, University of Calgary, Calgary, AB T2N 1N4 Canada; 5 Department of Physiology and Pharmacology, University of Calgary, Calgary, AB T2N 1N4 Canada; 6 Libin Cardiovascular Institute of Canada, University of Calgary, Calgary, AB T2N 1N4 Canada; 7 Centre for Bioengineering Research & Education, University of Calgary, 2500 University Drive NW, Calgary, AB T2N 1N4 Canada

**Keywords:** Shear stress, Epithelial, Bioreactor, Gene expression, Psoriasin, Breast cancer

## Abstract

**Introduction:**

S100 proteins are intracellular calcium ion sensors that participate in cellular processes, some of which are involved in normal breast functioning and breast cancer development. Despite several S100 genes being overexpressed in breast cancer, their roles during disease development remain elusive. Human mammary epithelial cells (HMECs) can be exposed to fluid shear stresses and implications of such interactions have not been previously studied. The goal of this study was to analyze expression profiles of S100 genes upon exposing HMECs to fluid flow.

**Methods:**

HMECs and breast cancer cell lines were exposed to fluid flow in a parallel-plate bioreactor system. Changes in gene expression were quantified using microarrays and qPCR, gene-gene interactions were elucidated using network analysis, and key modified genes were examined in three independent clinical datasets.

**Results:**

S100 genes were among the most upregulated genes upon flow stimulation. Network analysis revealed interactions between upregulated transcripts, including interactions between S100P, S100PBP, S100A4, S100A7, S100A8 and S100A9. Overexpression of S100s was also observed in patients with early stage breast cancer compared to normal breast tissue, and in most breast cancer patients. Finally, survival analysis revealed reduced survival times for patients with elevated expression of S100A7 and S100P.

**Conclusion:**

This study shows that exposing HMECs to fluid flow upregulates genes identified clinically to be overexpressed during breast cancer development, including S100A7 and S100P. These findings are the first to show that S100 genes are flow-responsive and might be participating in a fundamental adaptation pathway in normal tissue that is also active in breast cancer.

## Introduction

Overexpression of S100 genes have been implicated in a wide range of biological processes, including cell proliferation, differentiation, inflammation, angiogenesis, metastasis, and immune invasion.^6,25,51,80^ The S100 gene family consists of more than 21 members, distributed in a cell-specific, tissue-specific, and cell cycle-specific manner in humans and other vertebrates where they regulate cellular responses by acting as intracellular calcium ion sensors through interactions with a variety of cell-surface receptors such as RAGE, ALCAM, IL-10R, FGFR1 and TLR4.^1,4,6,11,24,51^ S100s, normally present in cells derived from the neural crest, macrophages, and epithelium,^18,51,63^ are known to be overexpressed in several types of cancers, including breast carcinomas.^3,23,84^ However, their roles during normal organ function and breast cancer development have not been elucidated.^6^

Luminal epithelial and myoepithelial cells in the mammary gland are potentially exposed to fluid forces resulting from interstitial flow, lymphatic drainage, and lactation.^72^ Although fluid flow is known to significantly alter gene expression patterns and phenotypic profiles in other types of cells,^66,71^ it is not known how fluid flow modulates breast epithelial cells. Magnitudes of cell surface shear stresses in the normal breast epithelium have not been defined; however, they are believed to be lower than those experienced in the breast tumor microenvironment, which are on the order of 0.01–0.7 Pa.^30,43,54^ During lactation, these magnitudes are probably higher due to elevated blood flow to the breast in response to increased cell proliferation and lobular expansion.^83^

The objective of this study was to evaluate the effect of fluid flow on gene expression in human mammary epithelial cells (HMECs), with emphasis on the S100 proteins, and comparison with expression in unstimulated and flow-stimulated breast cancer cell lines as well as in human tumor data. Differentially expressed genes were mapped onto a human interactome on the Cytoscape software platform and used to extract pronounced interactions between static and flow-exposed cells. Prognostic values of several overexpressed S100 genes were evaluated in expression profiles from two independent breast cancer clinical datasets. Finally, raw microarray data from a previous clinical study^39^ consisting 5 healthy and 9 ductal carcinoma *in situ* (DCIS) patient tissue samples was analyzed to identify S100 genes that are differentially expressed during the early stages of breast cancer development.

## Materials and Methods

### Cell Culture and Flow Experiments

HMECs and BT-474 breast cancer cells were respectively obtained from Lonza (Walkersville MD, USA) and ATCC (American Type Culture Collection, Manassas VA, USA), and cultured per recommendations. MCF-7 and SK-BR-3 cells were provided by Dr. Carrie Shemanko (Department of Biological Sciences, University of Calgary) and were grown in Dulbecco’s Modified Eagle’s Medium (Life Technologies, Inc., ON, Canada) supplemented with 10% fetal bovine serum, 1% l-glutamine and 1% penicillin–streptomycin. All cell culture procedures were conducted within a sterile biosafety cabinet using aseptic techniques. Cells were grown in culture flasks and allowed to expand by incubating at 37 °C and 5% CO_2_. Media was changed every 2 days until cells were about 70% confluent, at which point they were expanded or transferred to glass slides.

**Figure 1:**
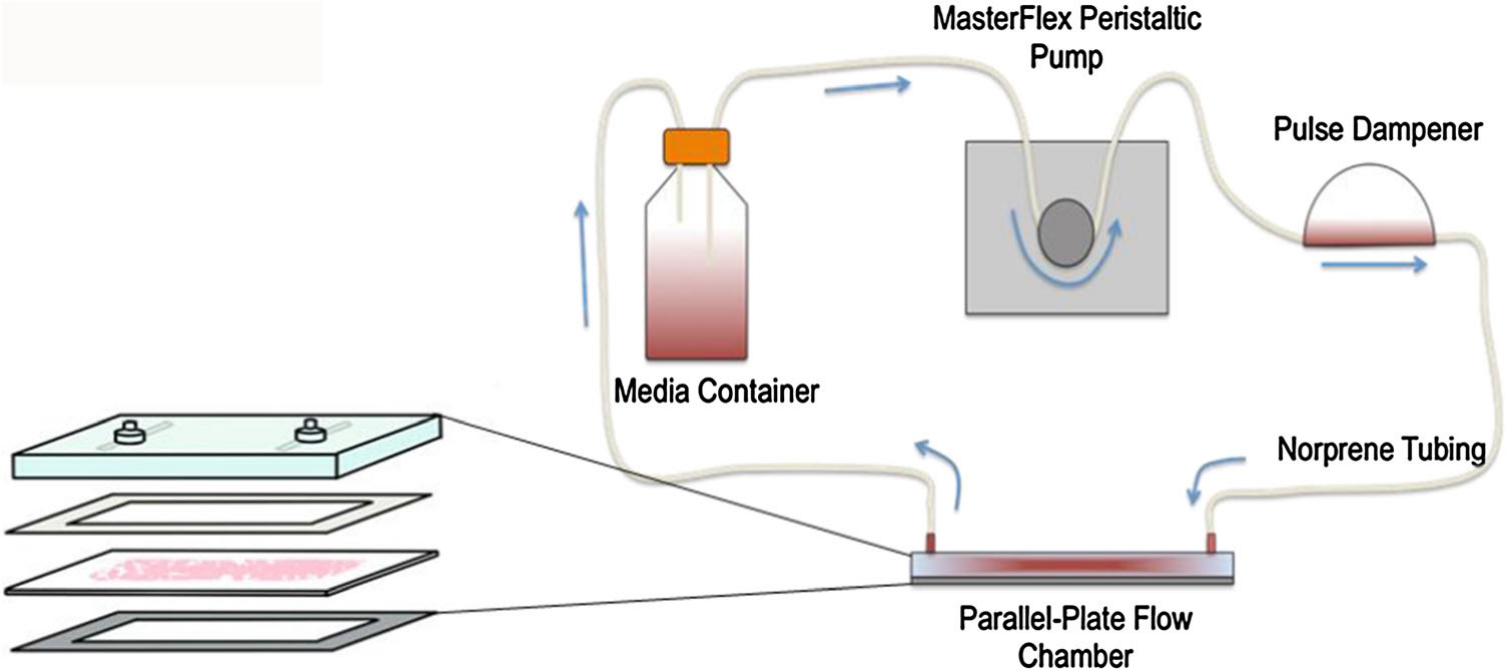
The parallel-plate bioreactor system used to expose cells to fluid flow. The volumetric flow rates of the flow medium corresponding to various magnitudes of shear stress on cell monolayers were determined using the Navier–Stokes equation for a Newtonian fluid in parallel plate geometry.^22,62,66^

For bioreactor culture, cell monolayers were cultured on glass slides pre-coated with 145 *µ*g/mL Rat Tail collagen I (Life Technologies, Inc., ON, Canada) and grown to confluence. Some slides were exposed to fluid flow by setting up the parallel-plate flow chamber as previously described,^66^ using MEGM cell culture media and with the addition of pulse dampeners to create steady flow. Figure 1 is an illustration of the parallel-plate bioreactor system. Cells were exposed to an average shear stress of 0.1 or 1 Pa for 20 h, and flow was provided by a Masterflex peristaltic pump and tubing (Cole Parmer, Montreal, QC, Canada).

### RNA Extraction and Quantification

Cells on glass slides exposed to flow or grown in static conditions were washed twice with cold PBS, trypsinized, scraped using a cell scraper, collected in appropriate centrifuge tubes, and stored on ice. Cells were then pelleted by spinning at 400 g for 4 min, and RNA was isolated using the EZNA Total RNA Kit (Omega Bio-Tek, Inc., Norcross, GA, USA) as per the manufacturer’s protocol. In summary, TRK Lysis Buffer was added to cell suspensions and thoroughly mixed to lyse cells. Lysates were then loaded into Omega Homogenizer Columns pre-inserted in 2 mL collection tubes, and centrifuged for 2 min at 20,000 × *g*. Next, 70% ethanol was added to the homogenized lysate, mixed thoroughly, loaded into HiBind® RNA Mini Columns pre-inserted in 2 mL collection tubes, and centrifuged at 10,000 × *g* for 60 s. HiBind® columns were then washed once with RNA Wash Buffer I and twice with RNA Wash Buffer II. Finally, DEPC Water was added to HiBind® columns and centrifuged at 20,000 × *g* for 2 min to elute RNA. Hydrated RNA samples were stored at – 80 °C until analysis.

RNA was assayed using the Quant-iT^TM^ RiboGreen RNA Assay Kit (Life Technologies, Inc., Burlington, ON, Canada) according to manufacturer’s instructions. Briefly, working solutions of TE Buffer, Quant-iT^TM^ RiboGreen RNA reagent, and standard ribosomal RNA were prepared and loaded into a 96 well microplate. Standard curves were generated by measuring sample fluorescence using a SpectraMax M2 microplate reader (Molecular Devices, Sunnyvale, CA, USA), with excitation and emission wavelengths set at 485 and 520 nm, respectively. Experimental RNA samples were prepared similarly, and unknown RNA concentrations were interpolated from standard curves using the SoftMax Pro software (Molecular Devices, Sunnyvale, CA, USA). All pipettes and working surfaces for RNA isolation and quantification were decontaminated using RNAse Away^TM^ Decontamination Reagent (Thermo Scientific, Wilmington, DE, USA).

### RT-qPCR

cDNA was synthesized using the qScript^TM^ cDNA Synthesis Kit (Quanta Biosciences, Gaithersburg, MD, USA) according to the manufacturer’s protocol. Briefly, 1000 ng of RNA was added to nuclease-free water, qScript Reaction Mix and qScript Reverse Transcriptase to a final volume of 20 *µ*L, vortexed gently and centrifuged for 10 s. The cDNA synthesis reaction was performed by incubating the mixture at 22 °C for 5 min, 42 °C for 30 min and 85 °C for 5 min using an MJ Mini Personal Thermal Cycler (Bio-Rad, Hercules, CA, USA).

For real time PCR amplification, a total reaction volume of 20 *µ*L was prepared by adding 2 *µ*L cDNA, 10 *µ*L TaqMan® Fast Advanced Master Mix (Thermo Fisher Scientific, Ottawa, ON, Canada), and 8 *µ*L mixture of primers, probes and nuclease-free water. Quantitative PCR was carried out on a ViiA 7 Real Time PCR System (Life Technologies, Foster City CA, USA). PCR experiments were run in triplicates and B2M was used as the reference gene. The following cycling conditions were employed for target gene amplification: 50 °C for 2 min, then 95 °C for 20 s, followed by 40 cycles of 1 s at 95 °C and 20 s at 60 °C. Relative gene expression was calculated using the comparative cycle threshold method.^49^ Statistical significance for differential expression of genes between conditions was determined using Student’s *t*-test.

### Cell Line Microarray Studies

GeneChip PrimeView Human Gene Expression Microarrays (Affymetrix, Santa Clara, CA, USA) were used to quantify changes in gene expression. For this, RNA from flow stimulated and unstimulated samples were fluorescently labeled and applied on the chip to allow for hybridization. Microarrays were run on RNA from a minimum of three replicates each for unstimulated and flow-stimulated cells. All microarray experiments were performed at the Arnie Charbonneau Cancer Institute Microarray Facility (Alberta Health Region, Canada).

Signal intensity files were pre-processed using robust multichip average normalization.^65^ Microarray data was analyzed using Biometric Research Branch—ArrayTools Version 4.5.0 (National Cancer Institute, USA)^60^ and Partek Genomics Suite Version 6.6 (Partek, Inc., Missouri, USA).^27,77^ Differentially expressed genes (fold change ≥ 2.0 and *p*-value ≤ 0.01) were identified by analyzing class comparisons between static and flow samples for each cell line, and for all static and flow-exposed breast cancer cells. Heatmaps were generated to show targets that are differentially expressed between unstimulated and flow-stimulated cells. Similarly, relative gene set expression analyses were performed against gene sets from the Molecular Signature Database (Broad Institute, USA), with at least five differentially expressed genes in a particular Gene Ontology category.^32,71,77^

### Ductal Carcinoma *In Situ* and Healthy Breast Tissue Microarray Studies

Gene expression profiles from 5 healthy breast tissue samples and 9 DCIS patients^39^ were analyzed to evaluate differential expression of genes during early stages of breast cancer development. Gene expression was quantified using Affymetrix oligonucleotide Human Genome U133 plus 2.0 Arrays and raw microarray files were downloaded from the NCBI microarray data bank (accession number GSE21422). Complementary RNA was amplified from 1 *μ*g of total RNA using the GeneChip® One-Cycle Target Labelling Kit (Affymetrix). Quantities of in vitro transcription and fragmentation products were assessed using the Agilent 2100 Bioanalyzer.^39^ Microarray data was pre-processed using the robust multichip average normalization method and patterns of expression analyzed using the Biometric Research Branch—ArrayTools software package, version 4.5.0 (National Cancer Institute, USA).^60^ Differentially expressed genes (fold change ≥ 2.0 and *p*-value ≤ 0.01) were identified by analyzing class comparisons.

### Interaction Network Analysis

Gene patterns from cell line microarray data were used to extract pronounced gene features and visualize gene interaction networks using network analysis on the Cytoscape (version 3.3) software platform as previously described.^50,75^ Differentially expressed genes between static and flow-exposed breast cancer cells were mapped onto a human interactome network obtained from integrated complex traits networks (iCTNet), version 2.0.^26,64,67,75^ The Advanced Network Merge tool was used to make a union merge of individual sub-networks and their differentially expressed first degree neighbours.^50,76^ Statistical significance threshold of differentially expressed genes in network analysis was set at fold change ≥ 2.0 and *p* < 0.05.

### Clinical Patient Data Analysis

Level 3 mRNA expression data from The Cancer Genome Atlas (TCGA) was obtained from Synapse ID: 1461151.^9^ Gene expression data was measured using the Illumina HiSeq 2000 RNA Sequencing version 2 analysis platform (RNA-Seq by Expectation Maximization). This dataset contains whole genome expression data for 104 healthy volunteers, 317 luminal A, 93 luminal B, 26 HER2 and 81 basal breast cancer patients. Breast cancer subtype classification was based on immunohistochemical expression of ER, PR and HER2.^29^

MATLAB 8.3 (MathWorks, Inc., Natick, MA, USA) was used to create box plots of relative expression levels of each target in breast cancer patients and healthy volunteers. The boxes showed the median and interquartile ranges, while whiskers showed the minimum and maximum expression levels for each target in each patient category. Statistical significance was determined using a two-sample *t*-test assuming equal means and equal, but unknown variances.

Prognostic value of selected genes was evaluated using Kaplan–Meier curves to compare relapse-free survival times in a breast tumor microarray data set containing 2878 breast cancer patients.^28^ Data was obtained from http://kmplot.com/analysis/ and statistical significance was determined using the log-rank test.

## Results

### Fluid Flow Affects Gene Expression in Human Mammary Epithelial Cells

**Figure 2:**
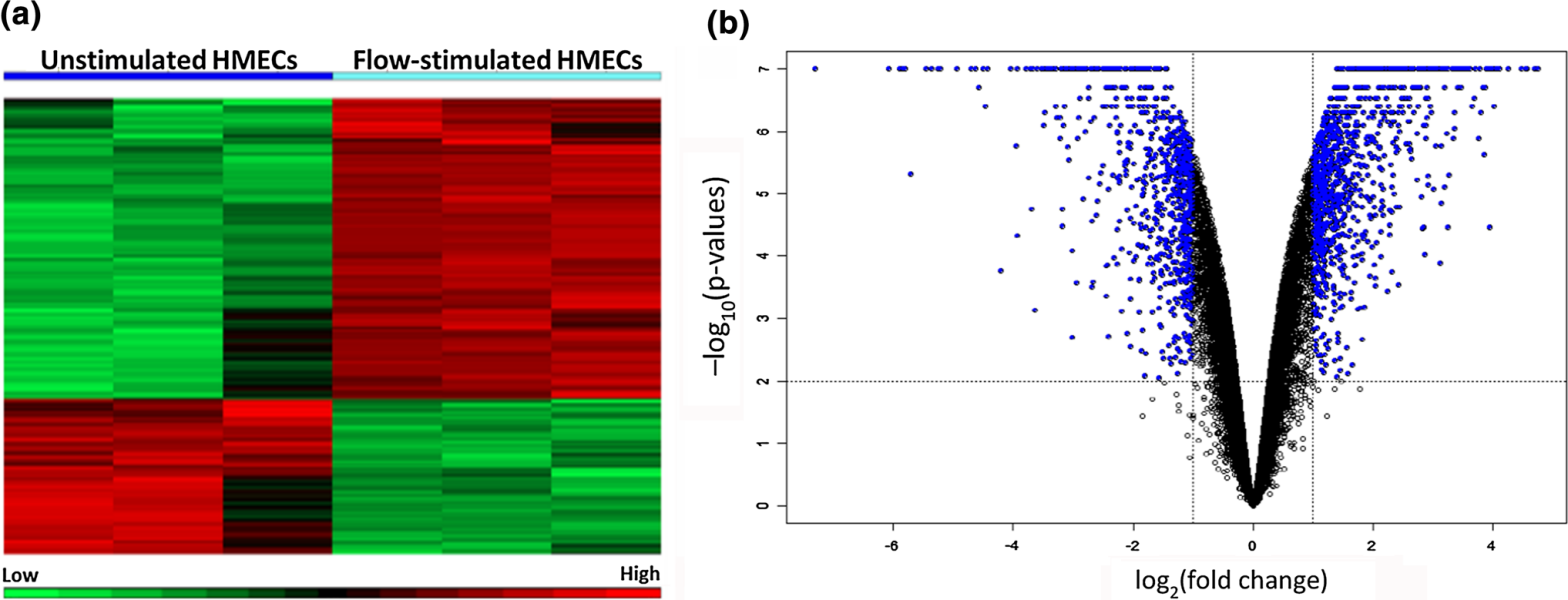
Fluid flow exposure affects HMEC gene expression. (**a**) Heat maps of differentially expressed genes between unstimulated and flow-stimulated HMECs. Three replicates were performed for each condition. Upregulated genes are indicated in red while downregulated genes are indicated in green. Genes that were not differentially expressed are indicated in black. (**b**) Volcano plot of genes analysed on microarrays. Blue and black dots represent differentially and insignificantly expressed genes, respectively. Genes with fold changes ≥ 2.0 and *p* ≤ 0.01 were considered differentially expressed.

To investigate the effect of fluid flow on HMECs, cultured cells were exposed to fluid flow in a parallel-plate bioreactor system and analyzed for changes in gene expression. Microarray data showed unique clusters of differentially expressed genes between flow-stimulated and unstimulated HMECs (Fig. 2a), with more than 1200 genes being significantly altered. All genes analyzed on the microarrays are represented in the volcano plot presented in Fig. 2b, with stringency levels of differential expression set at fold change ≥ 2.0 and *p*-values ≤ 0.01. Table 1 lists the most overexpressed genes in the flow-stimulated cells (only genes with fold changes greater than 5 are shown). This table consists three S100 genes, including S100P, S100A7 and S100A7A. One hundred and nine genes were downregulated at least 5-fold or more upon flow stimulation.

**Table 1:** List of genes upregulated more than 5-fold upon exposure of HMECs to fluid flow

Symbol	Fold change	Parametric *p*-value	Gene name
**S100A7**	33.41	8.43E−03	S100 calcium binding protein A7
SPRR1A	18.82	2.21E−02	Small proline-rich protein 1A
LCN2	16.7	1.38E−02	Lipocalin 2
PI3	15.38	7.95E−03	Peptidase inhibitor 3, skin-derived
SPRR2D	14.88	7.51E−03	Small proline-rich protein 2D
IL1F9	14.86	4.70E−05	Interleukin 1 family, member 9
IGFL1	14.2	4.24E−04	IGF-like family member 1
NCF2	13.87	3.21E−04	Neutrophil cytosolic factor 2
GLRX	12.78	1.35E−03	Glutaredoxin (thioltransferase)
**S100P**	11.91	2.74E−02	S100 calcium binding protein P
A2ML1	10.76	2.24E−02	Alpha-2-macroglobulin-like 1
CYP1A1	10.75	3.01E−03	Cytochrome P450, family 1, subfamily A, polypeptide 1
**S100A7A**	9.93	4.46E−02	S100 calcium binding protein A7A
CYP4F3	9.74	1.08E−05	Cytochrome P450, family 4, subfamily F, polypeptide 3
TMPRSS4	9.32	3.47E−02	Transmembrane protease, serine 4
SPP1	9.04	6.10E−06	Secreted phosphoprotein 1
CST6	8.11	2.45E−02	Cystatin E/M
NDRG4	7.97	1.50E−06	NDRG family member 4
IL8	7.88	9.00E−03	Interleukin 8
HMOX1	7.86	< 1e−07	Heme oxygenase (decycling) 1
ALDH3B2	7.72	4.13E−03	Aldehyde dehydrogenase 3 family, member B2
MAP3K8	6.89	5.28E−05	Mitogen-activated protein kinase kinase kinase 8
MAP2	6.32	4.20E−03	Microtubule-associated protein 2
FUT3	6.18	9.61E−03	Fucosyltransferase 3
SLC16A14	5.94	5.00E−07	Solute carrier family 16, member 14
BSPRY	5.92	1.73E−02	B-box and SPRY domain containing
TMPRSS11E	5.92	4.65E−03	Transmembrane protease, serine 11E
STEAP4	5.78	1.95E−02	STEAP family member 4
CLDN4	5.51	9.76E−03	Claudin 4
GUCY1B3	5.48	4.71E−02	Guanylate cyclase 1, soluble, beta 3
CFB	5.47	7.00E−06	Complement factor B
CYP4F11	5.08	1.13E−05	Cytochrome P450, family 4, subfamily F, polypeptide 11
AGR2	5.03	2.40E−06	Anterior gradient homolog 2 (*Xenopus laevis*)

S100 genes are shown in bold

Key molecular functions and biological processes significantly affected by flow exposure were identified using relative gene set comparison analysis of microarray data. The major functions and processes that were significantly affected by fluid flow exposure are involved in normal breast function and cancer development, including cellular response to TGF-β stimulus, regulation of epithelial to mesenchymal transition (EMT), cell adhesion, proliferation, differentiation and motility (Table 2). Threshold of relative gene subset enrichment was set at enrichment scores greater than 5 and *p*-values less than 0.01.

**Table 2:** List of selected molecular functions and biological processes significantly enriched by flow exposure^68^

Cellular function or process	Enrichment score	Enrichment *p*-value
Regulation of cell cycle process	14.8	7.0E−29
Cellular response to chemical stimulus	10.8	2.0E−05
Cellular response to growth factor stimulus	8.7	1.7E−04
Regulation of cell proliferation	7.6	5.1E−04
Positive regulation of metaphase/anaphase transition of cell cycle	7.2	7.8E−04
Cell differentiation involved in embryonic placenta development	7.0	6.1E−06
Cellular response to TGF-β stimulus	6.6	1.3E−03
Regulation of cell motility	5.9	5.2E−02
RNA splicing, *via* transesterification reactions	5.7	6.9E−09
Regulation of cell adhesion	5.4	4.4E−03
Positive regulation of stem cell differentiation	5.2	4.9E−09
Regulation of epithelial to mesenchymal transition	5.0	6.8E−03
Cyclin-dependent protein kinase holoenzyme complex	5.0	1.0E−04

Functions and processes with relative enrichment scores greater than 5 and *p*-values less than 0.01 were considered significant. Descriptions of molecular functions and biological processes were obtained from the Gene Ontology database of the Biometric Research Branch—ArrayTools software package (v4.5.0)^22^

### S100 Genes are Upregulated in Flow-Stimulated HMECs and Breast Cancer Cells

**Figure 3:**
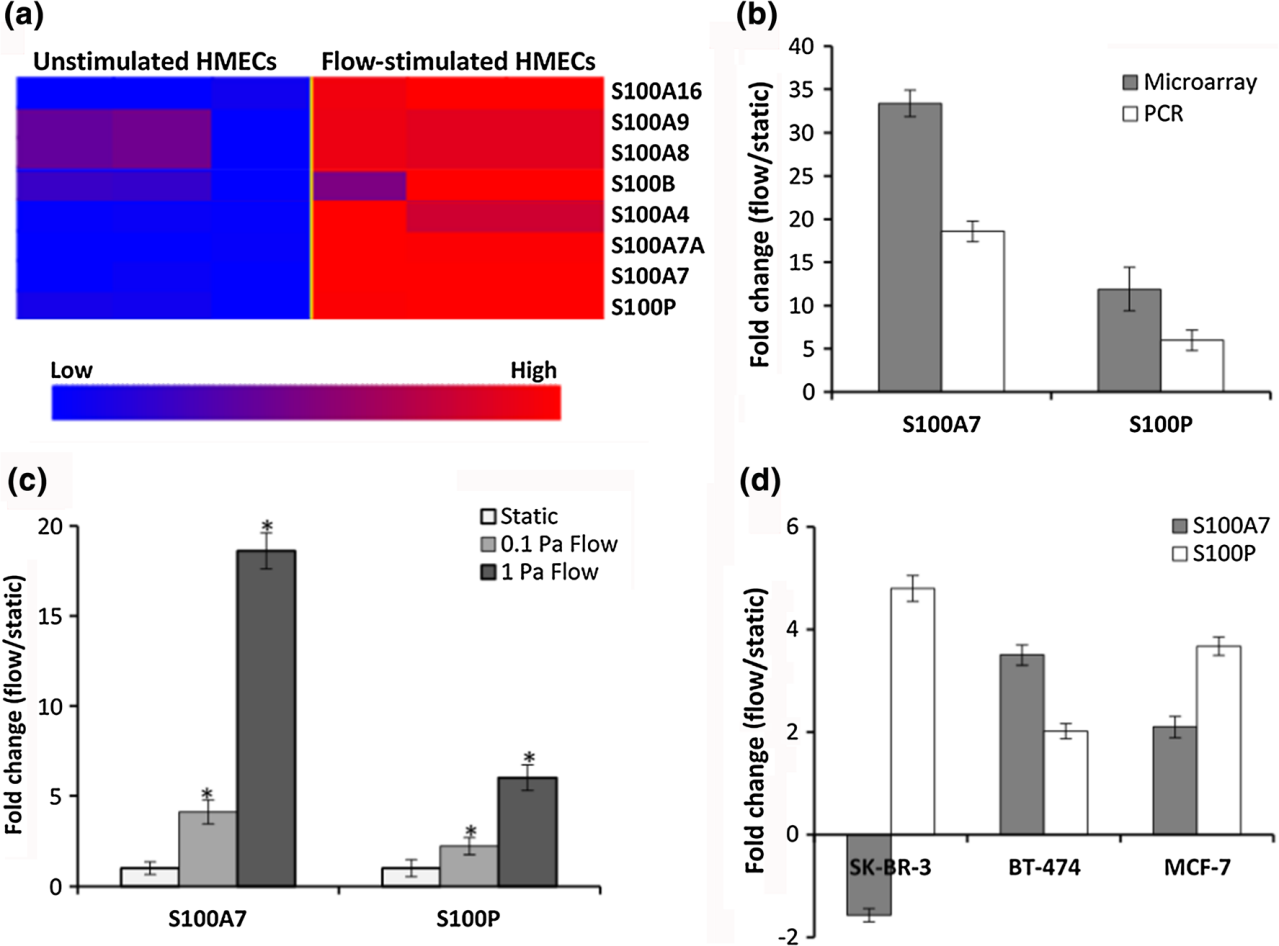
S100 genes are upregulated upon flow exposure. (**a**) Heat maps showing differentially expressed (fold change ≥ 2.0 and *p*-value ≤ 0.01) S100 genes in HMECs cultured under static and flow conditions; (**b**) S100A7 and S100P gene expression quantification by microarrays and quantitative PCR; (**c**) quantitative PCR analysis of S100A7 and S100P expression in HMECs grown in static culture or stimulated with fluid flow for 20 h at 0.1 and 1 Pa; and (**d**) Quantitative PCR analysis of S100A7 and S100P relative expression in SK-BR-3, BT-474 and MCF-7 breast cancer cells cultured in static conditions or exposed to 1 Pa shear stress. Fold changes were calculated using the comparative cycle threshold method and normalized to expression in static controls. Asterisks indicate statistically significant gene expression changes between static and flow-exposed cells (fold change ≥ 2; *p* < 0.01). Triplicate wells were run for every sample and B2M was used as the reference gene. Data shown represents the mean ± standard error of the means of data from at least three samples for each condition.

To examine which S100 genes were significantly altered upon flow stimulation, a gene set consisting all 21 S100 genes was created and used to analyze differential expression by class comparisons. The resulting heat map showed consistent upregulation of 8 of the 21 S100 genes upon flow exposure of HMECs (Fig. 3a). Quantitative PCR analysis showed that the relative expression of two of the most upregulated genes, S100A7 and S100P, followed the same trend as the microarray results (Fig. 3b).

Next, to investigate at what shear stress S100A7 and S100P would be significantly upregulated following flow stimulation, HMECs were exposed at 0.1 Pa flow derived from the parallel-plate bioreactor system, and relative expression compared to expression in cells exposed at 1 Pa flow. Exposure times for both shear stresses were 20 h. As shown in Fig. 3c, both S100A7 and S100P are significantly upregulated at 0.1 Pa (4.1 and 2.2-folds, respectively) and are more overexpressed at 1 Pa (18.6 and 6.0-folds, respectively).

To investigate the relevance of our findings in breast cancer, we exposed SK-BR-3, BT-474 and MCF-7 breast cancer cells to fluid flow at 1 Pa derived from the parallel-plate bioreactor system, and analyzed changes in gene expression by quantitative PCR. These cell lines represent HER2+, luminal B and luminal A breast cancer, respectively.^9^ Patients with each subtype present with distinct histological characteristics and clinical outcomes.^9,69^ Interestingly, S100A7 was overexpressed in two of the cell lines while S100P was upregulated in all three cell lines upon flow stimulation (Fig. 3d), suggesting a potential role during breast cancer progression.

### Network Analysis Revealed Key Genes Involved in Cell Flow Responses

To visualize molecular interaction networks between upregulated S100 genes, gene expression data was imported and analyzed on the Cytoscape software platform. A highly curated human interactome network was downloaded using iCTNet version 2.0.^67^ The protein network corresponding to the differentially expressed genes between unstimulated and flow-stimulated HMECs (with fold changes ≥ 5) and their first-degree neighbours was highly clustered, indicating a high degree of modularity of gene networks when HMECs are exposed to flow.

**Figure 4:**
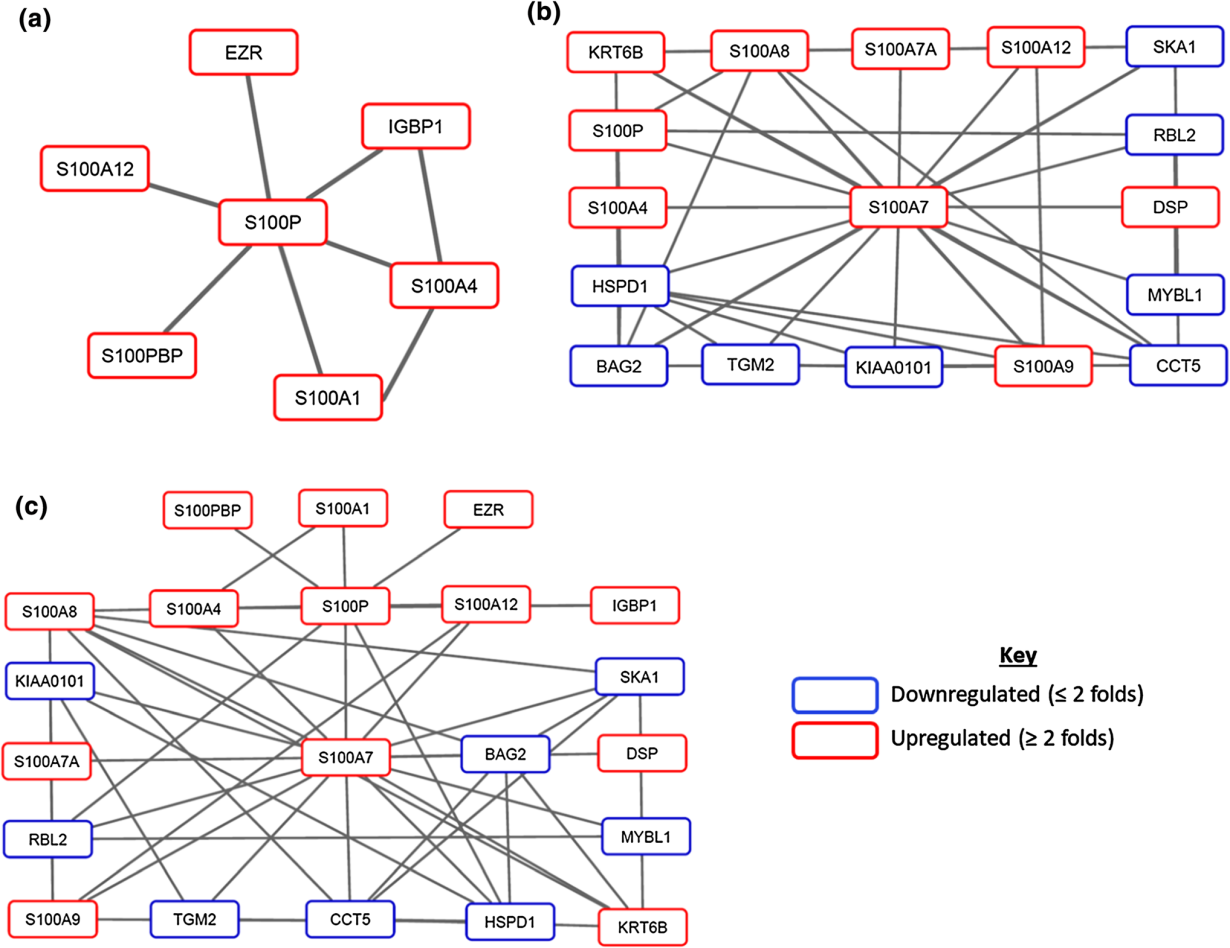
Network analysis revealed interactions between S100 genes. Sub-networks of S100P (**a**) and S100A7 (**b**) and their differentially expressed first degree neighbours. (**c**) Merged network of S100P and S100A7 sub-networks, created using the Advanced Network Merge tool in Cytoscape. Nodes with red and blue borders represent genes that were upregulated and downregulated upon flow exposure, respectively. Statistical threshold of differential expression for network analysis was set for genes with fold change ≥ 2 and *p* < 0.05.

Next, interaction sub-networks of the two most upregulated S100 genes, S100P and S100A7, were analyzed to better understand functional links through which flow-induced overexpression potentially alters cellular events and phenotypic profiles in HMECs. Interactions of S100P and its differentially expressed first degree neighbours, including S100PBP, S100A1, S100A4, S100A12, EZR and IGBP1, were observed (Fig. 4a). The sub-network for S100A7 was more clustered and revealed interactions between S100A7 and other S100 genes, such as S100P, S100A4, S100A7A, S100A8, S100A9 and S100A12, as well as with non-S100 genes such as KRT6B, TGM2, HSPD1, CCT5 and RBL2 (Fig. 4b). Co-expression of S100A8, S100A9 and S100A12 has been reported in another study.^10^ A union merge of the two sub-networks was created using the Advanced Network Merge tool in Cytoscape. As shown in Fig. 4c, the merged network reveals potential interactions through which overexpression of S100P and S100A7 potentially contributes to biological processes, such as through the upregulated S100PBP-S100P-S100A7-S100A9 axis.

### S100 Genes are Differentially Expressed During Early Stages of Breast Cancer Development

**Figure 5:**
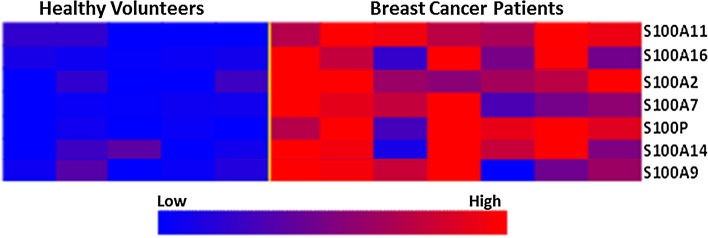
S100 genes are upregulated during early stages of breast cancer development. Heat maps showing differential expression of S100 genes and metalloproteinases between healthy breast samples and ductal carcinoma *in situ* (DCIS) patients. Upregulated and downregulated genes are indicated in red and blue, respectively. Raw microarray files were obtained from GSE21422 (Kretschmer *et al.* 2011). Statistically significant genes were those with fold changes ≥ 2 and *p* ≤ 0.01.

To investigate the relevance of our findings during early stages of breast cancer development, differential expression of S100 genes was analysed using data from a previous microarray study.^39^ This study contained microarray data from 5 healthy breast and 9 DCIS patient tissue samples. Interestingly, S100P and S100A7 were among the most upregulated S100 genes in patient tissues, with fold changes of 30.3 and 25.4, respectively. Other significantly overexpressed S100 genes and their fold changes include S100A14 (11.9), S100A2 (5.0), S100A16 (2.6), S100A9 (2.4) and S100A11 (2.1). Figure 5 shows the heat map of differential expression of S100 genes between tissue samples from healthy volunteers and DCIS patients.

### Expression Profiles of S100P and S100A7 in Clinical Datasets

**Figure 6:**
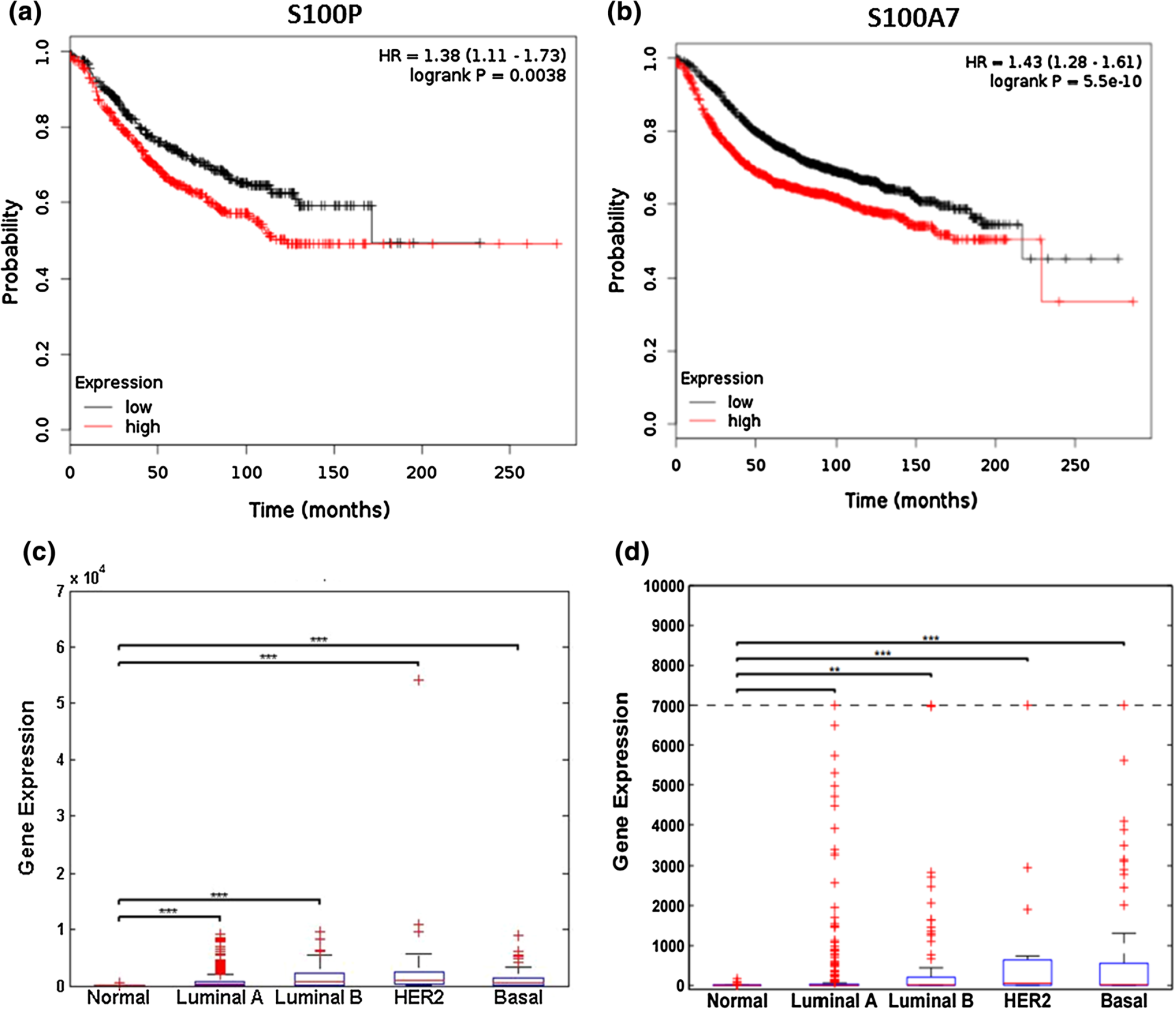
Relative expression of S100P and S100A7 between healthy volunteers and breast cancer patients. Kaplan–Meier plots showing relapse-free survival analysis of breast cancer patients with lymph node positive status (*n* = 936), stratified by S100P (**a**) and S100A7 (**b**) expression. Data was obtained from http//kmplot.com/analysis and statistical significance was determined using the log-rank test. Box plots showing expression of (**c**) S100P and (d) S100A7 between healthy volunteers (normal) and patients with several stratifications of breast cancer. Expression data was obtained from TCGA and consisted of 104 healthy volunteers, 317 luminal A, 93 luminal B, 26 HER2 and 81 basal patients. **p ≤ 0.01; ***p ≤ 0.001.

To evaluate the relevance of our findings in breast cancer diagnosis, we evaluated the prognostic value of S100P and S100A7 in a microarray data set of breast tumors from 1809 patients.^28^ Kaplan–Meier curves of relapse-free survival times of breast cancer patients with lymph node positive status (*n* = 936), stratified by S100P (Fig. 6a) and S100A7 (Fig. 6b) expression levels, showed patients with higher expression levels (red lines) have reduced survival times compared to patients presenting with lower expression (black lines). The median value of expression was used as the cut off for low and high expression.

Finally, we evaluated expression of S100P and S100A7 in gene expression data from 517 patients presenting with the various molecular subtypes of breast cancer compared to expression in 104 healthy volunteers. We observed overexpression of S100P in patients with all subtypes of breast cancer (Fig. 6c) and overexpression of S100A7 in patients with luminal B, HER2 positive and basal breast cancer (Fig. 6d).

## Discussion and Conclusions

In this study, we found that fluid flow alters expression of many genes in mammary epithelial cells, and that some of these genes, especially those in the S100 family, appear to be important in breast cancer. S100 proteins are small molecular EF-hand calcium-binding proteins found in a broad array of tissues with a variety of expression levels and several members have been implicated in breast cancer development and progression.^1,7,55,70,82^ Breast cancer is usually caused by genetic mutations that bestow epithelial cells lining breast ducts with the ability to grow abnormally.^16^ Development and progression of this complex disease involves mechanical interactions between normal epithelial cells and associated microenvironments, including exposure to fluid flow from blood supply and lymphatic drainage.^16,30,78^ This study is the first to investigate the potential implications of fluid flow exposure on gene expression profiles of HMECs for the purpose of identifying potential drivers of disease development.

Consistent overexpression of S100 genes was observed upon flow stimulation of HMECs, with two S100 genes, S100A7 and S100P, among the most upregulated. Neither of these genes have been previously identified as flow responsive. However, both have been shown to be involved in cell adhesion, motility, inflammation, and proliferation—cellular processes affected by fluid flow in other cell types.^2,23,26,37^ Besides playing a putative role in breast cancer progression,^40^ S100A7 (psoriasin) influences cytokine production and affects cell recruitment, supporting previous studies that revealed a potential role in infection resistance and immunity in tissues that produce elevated levels of this protein.^17,38,79^ Interestingly, S100A7 is also upregulated during lactation,^61^ suggesting it may play a role in breast tissue remodelling during pregnancy. S100A19, the ancestor of S100A7, is expressed differentially during different stages of mammary gland development,^41^ supporting the position that S100A7 might play a role during breast development. S100P, on the other hand is present in several adult tissues and known to participate in epithelial differentiation in the esophagus, duodenum, and prostate.^59^ Our findings reveal potential roles of S100A7 and S100P in regulating cellular events in the healthy mammary epithelium.

Though comparison of the response to fluid shear between normal and cancer cells was not a primary objective of the study, it can be observed in Fig. 3 that the effect of flow on expression of the S100 targets is greater in the normal cells. This is an interesting observation that can easily be associated to the normal epithelial and cancer cells being at different stages of the disease development spectrum. We have not generated any data to support the clinical relevance of these differences in the context of disease progression and submit that such a study would be complex and involve a broader representation of normal breast epithelial and cancer classifications.

Our results reveal differential expression of S100A7 and S100P in flow-stimulated breast cancer cell lines and in clinical populations. S100A7 was upregulated in two of the cell lines upon flow stimulation while S100P was upregulated in three breast cancer cell lines, suggesting potential roles in breast cancer. Overexpression of S100A7 and S100P was also observed in patients with most subtypes of breast cancer and survival plot analyses revealed reduced survival times for patients with elevated expression of both genes. These genes have previously been implicated in development of various malignancies, including those of the breast,^1,21,25,57^ colon,^35^ pancreas,^1^ lung,^48,57^ colorectal^19^ and cervix.^23,80^ Whereas S100A7 is known to be overexpressed in high grade pre-invasive breast cancer,^6,21,57^ S100P contributes to tumor growth, invasion, and metastasis either through extracellular binding to receptor for advanced glycation end products (RAGE) or intracellular coupling with cytoskeletal ezrin.^2,5,14,41,57^ Our findings support this position and reveal other S100 genes, including S100A9, S100A11, S100A14 and S100A16, whose expression levels are also altered during the early stages of breast cancer development.

Network analysis revealed key interactions through which flow-induced overexpression of S100A7 and S100P potentially contribute to modulation of normal breast epithelium and breast cancer development. We observed an interaction between S100P and its binding partner, S100PBP, a protein regulated by cathepsin Z and integrin *α*v*β*5 during S100P-mediated cell adhesion.^59,20,42,47^ S100P also interacts with other S100 genes such as S100A1, S100A4 and S100A12. S100A7 interacts with S100P as well as with other S100 genes including S100A4, S100A8, S100A9, S100A12 and S100A7A, an important paralog of S100A7 that is also involved in progression of psoriasis.^25,36^ S100A4 has been extensively evaluated as a prognostic and diagnostic biomarker in a broad array of cancers including breast,^1,13,52^ lung,^1,45^ pancreatic,^31,33,44^ liver,^46^ colorectal,^1,12^ melanoma,^1,81^ and mesothelioma.^56^ S100A8 and S100A9 usually form a heterodimer complex (S100A8/A9) that binds to TLR4 and plays an important role in the ‘seed and soil’ theory of cancer metastasis by binding to individual S100 soil sensor receptors and promoting cellular processes such as epithelial–mesenchymal transition, cellular motility and cancer cell invasiveness.^53,73,74,85^ Several genes in the sub-network of S100A7 were observed to be downregulated upon flow stimulation such as HSPD1, CCT5 and RBL2. HSPD1 and CCT5 are members of the family of molecular chaperones which participate in innate immune system signaling, supporting the role of S100A7 in infection resistance and immunity.^15,17,58^ The merged interaction network revealed interactions through which overexpression of S100A7 and S100P potentially contribute to altered cellular phenotypes through target regulation. Taken together, these findings support a recent report by Xia *et al.*^80^ implicating cell stress and inflammation to induction of S100 proteins in the acellular compartment where they bind to cell surface receptors and activate intracellular several signaling pathways.^26,34,80^

Overexpression of several S100 genes, including S100A7 and S100P in DCIS patients suggests possible roles during breast cancer development and progression. Kaplan–Meier curves revealed reduced survival times for patients with elevated expression of S100A7 and S100P, supporting previous results by Zhang *et al*.^84^ in which the prognostic value of all S100 genes were evaluated in breast cancer patients. Further, genome-wide analysis in patients revealed increased expression of S100A7 in luminal B, HER2 and basal breast cancer patients, and S100P in patients presenting with all subtypes of breast cancer. These findings are supported by previous publications in which S100 proteins are closely associated to tumorigenesis, cancer metastasis, changes in the tumour microenvironment, maintenance of pluripotency and potential implications as biomarkers and prognostic factors.^1,3,8,11,26,73,80,84^ More recently, Cancemi *et al.*,^8^ used a multi-omics analysis approach to support this position by showing that elevated expression of S100 proteins, including S100P and S100A7 in breast cancer patients correlate highly with reduced survival rates and occurrence of more aggressive disease subtypes.

In conclusion, this study shows that exposing HMECs to fluid flow upregulates genes clinically identified to be overexpressed in breast cancer patient profiles, including S100P and S100A7. Although we demonstrate here that fluid flow stimulation alters expression profiles of S100 genes, the activated signaling cascades and underlying mechanisms through which fluid flow exposure contributes to normal breast functioning and cancer progression remain elusive. Also, the functional implications of flow-induced upregulation of S100 proteins on cellular events and phenotypic profiles have not been investigated. Further experiments are therefore required to enhance our understanding of molecular pathways and affected functional events through which flow-induced upregulation of S100 proteins modulates normal breast epithelium and/or contributes to breast cancer development. The findings reported herein provide a critical foundation to such investigations, by suggesting that S100P and S100A7 may be important in health and disease. The results also demonstrate that our bioreactor platform is a useful tool for identifying how mammary epithelial cell exposure to fluid flow affects early stages of breast cancer development.
